# Efficacy of laser interstitial thermal therapy (LITT) for newly diagnosed and recurrent *IDH* wild-type glioblastoma

**DOI:** 10.1093/noajnl/vdac040

**Published:** 2022-04-06

**Authors:** John F de Groot, Albert H Kim, Sujit Prabhu, Ganesh Rao, Adrian W Laxton, Peter E Fecci, Barbara J O’Brien, Andrew Sloan, Veronica Chiang, Stephen B Tatter, Alireza M Mohammadi, Dimitris G Placantonakis, Roy E Strowd, Clark Chen, Constantinos Hadjipanayis, Mustafa Khasraw, David Sun, David Piccioni, Kaylyn D Sinicrope, Jian L Campian, Sylvia C Kurz, Brian Williams, Kris Smith, Zulma Tovar-Spinoza, Eric C Leuthardt

**Affiliations:** Department of Neuro-Oncology, UCSF Weill Institute for Neurosciences, San Francisco, California,USA; Department of Neurosurgery, Washington University School of Medicine, St. Louis, Missouri, USA; Department of Neurosurgery, The University of Texas MD Anderson Cancer Center, Houston, Texas, USA; Department of Neurosurgery, Baylor College of Medicine, Houston, Texas, USA; Department of Neurosurgery, Wake Forest Baptist Health, Winston-Salem, North Carolina, USA; Department of Neurosurgery, Duke University Medical Center, Durham, North Carolina, USA; Department of Neuro-Oncology, The University of Texas MD Anderson Cancer Center, Houston, Texas, USA; Department of Neurosurgery, University Hospitals—Cleveland Medical Center & Seidman Cancer Center, Cleveland, Ohio, USA; Department of Neurosurgery, Yale School of Medicine, New Haven, Connecticut, USA; Department of Neurosurgery, Wake Forest Baptist Health, Winston-Salem, North Carolina, USA; Department of Neurosurgery, Cleveland Clinic Lerner College of Medicine at CWRU, Cleveland, Ohio, USA; Department of Neurosurgery, NYU Grossman School of Medicine, New York, New York, USA; Department of Neuro-Oncology, Wake Forest Baptist Health, Winston- Salem, North Carolina, USA; Department of Neurosurgery, University of Minnesota Medical Center, Minneapolis, Minnesota, USA; Department of Neurosurgery, Icahn School of Medicine at Mount Sinai, New York, New York, USA; Department of Neuro-Oncology, Duke University Medical Center, Durham, North Carolina, USA; Department of Neurosurgery, Norton Neuroscience Institute, Louisville, Kentucky, USA; Department of Neuro-Oncology, University of California San Diego Health, La Jolla, California, USA; Department of Neuro-Oncology, Norton Neuroscience Institute, Louisville, Kentucky, USA; Department of Neuro-Oncology, Mayo Clinic, Rochester, Minnesota, USA; Department of Neuro-Oncology, NYU Langone Perlmutter Cancer Center, New York, New York, USA; Department of Neurosurgery, University of Louisville Health, Louisville, Kentucky, USA; Department of Neurosurgery, Barrow Neurological Institute, Phoenix, Arizona, USA; Department of Neurosurgery, SUNY Upstate Medical University, Syracuse, New York, USA; Department of Neurosurgery, Washington University School of Medicine, St. Louis, Missouri, USA

**Keywords:** high-grade glioma, *IDH* wild-type WHO grade 4 glioblastoma, laser interstitial thermal therapy (LITT), primary brain tumor, stereotactic laser ablation (SLA)

## Abstract

**Background:**

Treatment options for unresectable new and recurrent glioblastoma remain limited. Laser ablation has demonstrated safety as a surgical approach to treating primary brain tumors. The LAANTERN prospective multicenter registry (NCT02392078) data were analyzed to determine clinical outcomes for patients with new and recurrent *IDH* wild-type glioblastoma.

**Methods:**

Demographics, intraprocedural data, adverse events, KPS, health economics, and survival data were prospectively collected and then analyzed on *IDH* wild-type newly diagnosed and recurrent glioblastoma patients who were treated with laser ablation at 14 US centers between January 2016 and May 2019. Data were monitored for accuracy. Statistical analysis included individual variable summaries, multivariable differences in survival, and median survival numbers.

**Results:**

A total of 29 new and 60 recurrent *IDH* wild-type WHO grade 4 glioblastoma patients were treated. Positive *MGMT* promoter methylation status was present in 5/29 of new and 23/60 of recurrent patients. Median physician-estimated extent of ablation was 91%-99%. Median overall survival (OS) was 9.73 months (95% confidence interval: 5.16, 15.91) for newly diagnosed patients and median post-procedure survival was 8.97 months (6.94, 12.36) for recurrent patients. Median OS for newly diagnosed patients receiving post-LITT chemo/radiation was 16.14 months (6.11, not reached). Factors associated with improved survival were *MGMT* promoter methylation, adjuvant chemotherapy within 12 weeks, and tumor volume <3 cc.

**Conclusions:**

Laser ablation is a viable option for patients with new and recurrent glioblastoma. Median OS for *IDH* wild-type newly diagnosed glioblastoma is comparable to outcomes observed in other tumor resection studies when those patients undergo radiation and chemotherapy following LITT.

**Key Points**

- For glioblastoma patients ineligible for open resection, LITT is an effective option.
- For newly diagnosed patients, LITT is most effective when followed by radiation and chemotherapy.

**Importance of the Study**

Despite decades of research, limited options are available for the treatment of newly diagnosed and recurrent glioblastoma. Laser interstitial thermal therapy (LITT) is a valuable tool available to neurosurgeons to thermally ablate tumors in patients ineligible for conventional resection. This study reports the benefits of LITT in the largest series to date of molecularly defined *IDH* wild-type newly diagnosed and recurrent glioblastoma patients from a multicenter, prospective registry. Importantly, in patients with newly diagnosed and recurrent glioblastoma, median overall survival was comparable to historical controls treated with conventional approaches, especially when followed with chemoradiation for the newly diagnosed cohort. This is the first publication to focus exclusively on molecularly defined glioblastoma subgroups demonstrating safety and efficacy compared to historical benchmarks.

Isocitrate dehydrogenase 1 (*IDH1*) wild-type glioblastoma is the most common and aggressive primary brain malignancy in adults and accounts for 90% of high-grade glioma cases.^1^ Despite the best available treatments, patients inevitably progress and die from the disease. With standard of care therapy, median overall survival (OS) is estimated at 15-18 months with fewer than 10% of patients alive at 5 years.^2,3^ Extent of surgical resection (EOR) is an important clinical prognostic variable with studies showing initial gross total resection (GTR) improves OS.^4,5^ Unfortunately, not all tumors are amenable to conventional surgical resection at the time of diagnosis with only about 1/3 of patients able to achieve a GTR^6^ and 15%-25% of patients only able to receive biopsy.^7,8^ At the time of tumor progression, 80% of patients recur within 2-3 cm of the original surgical resection.^9^ Many patients at large academic centers are referred for re-resection for both diagnostic and therapeutic purposes,^10^ although the magnitude of benefit of re-resection may be restricted to a subset of patients. MRI-guided laser interstitial thermal therapy (LITT) is an FDA-cleared minimally invasive technique designed to safely ablate abnormal neurological tissue, including brain tumors, epileptic foci, and radiation necrosis.^11,12^ LITT offers a therapeutic alternative for patients with newly diagnosed and recurrent glioblastoma for whom conventional, open surgical approaches are not deemed optimal, whether due to surgical risk or patient preference.

The efficacy of LITT for the management of primary and metastatic brain tumors has not been evaluated in randomized clinical trials because of challenges with patient enrollment^13^ and the commercial availability of the product outside of clinical studies. Numerous retrospective studies have reported the utility of LITT for the management of newly diagnosed and recurrent glioblastoma. These studies have demonstrated strong safety data with variable efficacy in subgroups of patients. For example, some studies have demonstrated the association between extent of ablation (EOA) and improved survival in newly diagnosed unresectable glioblastoma,^14–16^ whereas others have not.^17^ However, limited patient numbers and mixing of patients with different histologic, molecular, and disease stages limits the ability to extract consistent conclusions. To overcome the limitations of prior studies, we performed an in-depth analysis of patients with newly diagnosed and recurrent *IDH* wild-type glioblastoma treated with LITT to better understand the optimal timing and integration of this therapy into the treatment of molecularly defined glioblastoma. A comprehensive analysis was performed from prospective multicenter data collected via the LAANTERN (Laser Ablation of Abnormal Neurological Tissue Using Robotic NeuroBlate System) registry (NCT02392078).

## Materials and Methods

### Patient Enrollment

LAANTERN is an institutional review board (IRB)-approved, multisite, prospective registry currently enrolling across 28 institutions in the United States. LAANTERN allows for follow-up data to be captured for up to 5 years following the LITT procedure. Fourteen institutions enrolled subjects eligible for this analysis. Details pertaining to the LAANTERN study were previously described.^11,12,18,19^ Monitoring with source verification and data management were enacted to ensure the accuracy of the deidentified data into the electronic database. The study protocol was reviewed and approved by the IRB at each participating center. Informed consent was obtained for all subjects using IRB-approved documentation.

**Figure 1.**
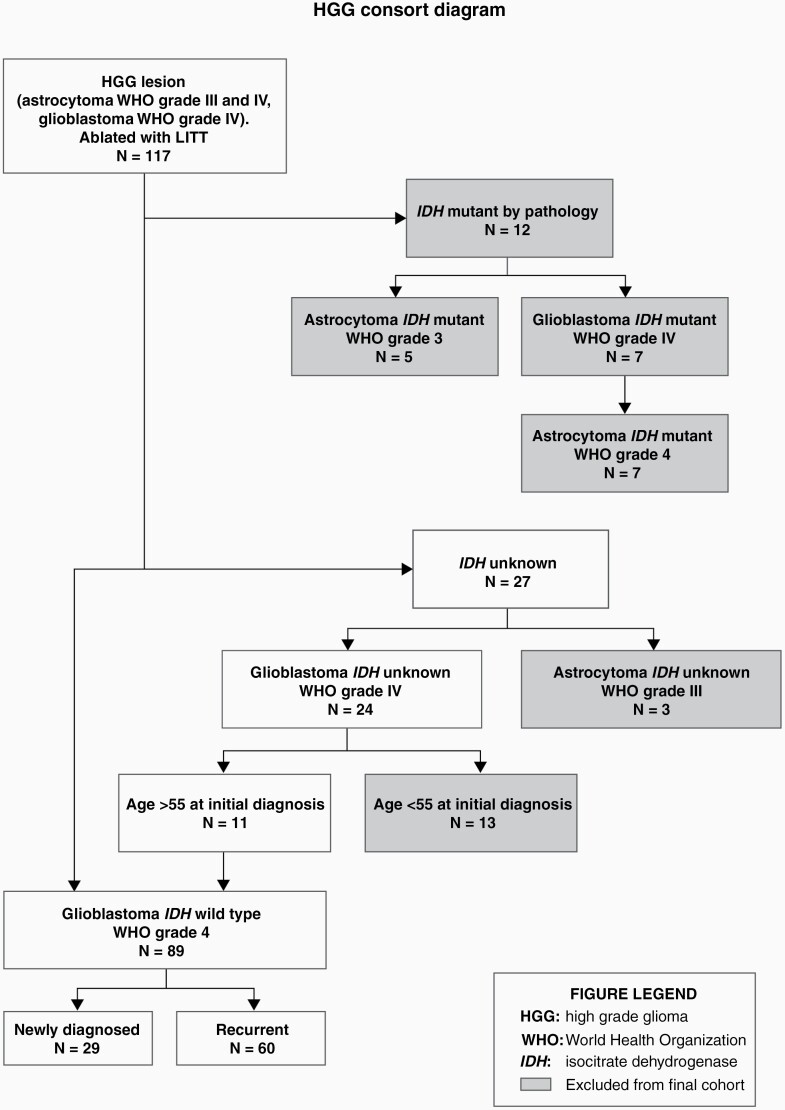
Consort diagram—cohort classification. Cohort designation per the latest WHO 2021 guidelines as well as inclusion/exclusion criteria for analysis are displayed in a flow chart. Those ultimately excluded from the final cohort analysis are shown in a shaded box.

A total of 117 subjects enrolled in LAANTERN met the following criteria: patients with a high-grade glioma lesion ablated with LITT with a biopsy-proven diagnosis of high-grade astrocytoma or glioblastoma; with biopsy at the time of LITT taking precedence over prior diagnosis. Patients were also required to be eligible for a 2-year follow-up. All patients in the cohort were consented for enrollment, prior to the procedure, in a consecutive series at each institution, between January 2016 and May 2019. Molecular markers were collected from biopsy pathology when available. Subjects were then separated into subgroups to isolate subjects with *IDH* status. Patients were divided into newly diagnosed and recurrent glioblastoma (see Figure 1 for a detailed description of patient selection). *IDH* status was determined from the pathology report and was available on 78 subjects. *IDH* wild-type subjects were reclassified into the glioblastoma WHO grade 4 category and subjects previously considered *IDH* mutant glioblastoma WHO grade IV were reclassified into astrocytoma WHO grade 4 based on the new WHO 2021 criteria.^20^ Subjects histologically designated as glioblastoma, but whose *IDH* status was unknown, were stratified by age at the time of initial diagnosis; patients with age >55 years were reclassified as *IDH* wild-type glioblastoma WHO grade 4 (n = 11).^21^ Subjects whose age was <55 years at the time of initial diagnosis, and who were without an *IDH* designation, were categorized as *IDH* unknown and not included in the analysis. The final analysis included 89 patients, including 29 newly diagnosed and 60 recurrent *IDH* wild-type glioblastoma (Figure 1).

### Surgical Management

All centers used the FDA-cleared NeuroBlate System (Monteris Medical, Minneapolis, MN, USA) as previously described.^22^ Surgical preplanning, technique, and biopsy during the procedure were performed as standard of care at each institution.

### Variables Collected

The LAANTERN registry collects demographic and health history information as well as disease-specific outcome measures. The following variables were collected and included in this analysis: demographics, diagnosis date, treatment prior to and after LITT, tumor location, tumor size, *IDH* mutation status, *MGMT* promoter methylation status, surgical “skin to skin” time, total laser ablation time and total energy applied to the lesion during ablation, adverse events (AEs), hospitalization data, OS data, progression-free survival (PFS) data, and Karnofsky Performance Scale (KPS) over time.

For the analysis of treatments received following LITT, a time parameter of 12 weeks was selected. The registry captured if a patient started chemotherapy and/or radiation but it was not known if they completed the entire course.

Survival data were estimated using the Kaplan-Meier method.^23^ OS was defined as the time from histopathologic diagnosis to death. PFS was defined as the time from the date of the LITT procedure to progression of the disease as defined by investigator-determined radiographic progression on MRI. Post-procedure OS was defined as the time from LITT procedure to death. KPS scoring was collected at baseline and at each follow-up visit.

AEs were reviewed by an independent safety committee composed of neurosurgeons with laser ablation expertise. The committee adjudicated reported AEs into neurological vs non-neurological categories and neurological deficits were specified as temporary or permanent. Relatedness to the NeuroBlate system, LITT procedure, and surgical procedure itself was also adjudicated. Events were rated as mild, moderate, or severe.

Deidentified MR images were collected at baseline (most immediately prior to procedure), procedure (intraoperative imaging), and discharge (1- to 3-day post-procedure), per standard practice at each institution. Available scans were sent for independent review and core laboratory analysis (Medical Metrics, Houston, TX, USA) to determine ablated lesion location, lesion volume, and EOA. Pre-procedure and comparative post-procedure/discharge MRI scans were required to complete per-patient analysis.

### Statistical Analysis

Categorical variables were summarized using relative frequencies and percentages. Continuous variables were summarized using mean ± standard deviation, median (25th, 75th percentile), and minimum and maximum. Variables were summarized separately for subjects with new tumors and subjects with recurrent tumors.

All variables were summarized at the individual level, except for lesion-specific variables including lesion depth, volume, and anatomical location, which were summarized at the lesion level. Molecular marker results from the procedure biopsy were used if available. If unavailable from the procedure biopsy, results from the baseline biopsy were used.

Median survival and Kaplan-Meier product limit analyses were used to depict the survival of patients. Log-rank tests were used to assess if survival or freedom from disease progression differed between those with new and recurrent lesions. For KPS, the *P*-value from the Student’s *t* test was used in a per-patient analysis to compare scores from each independent follow-up timepoint to baseline scores. A Cox proportional hazards model was used to assess multivariate differences in survival after the procedure based on these variables: new or recurrent glioblastoma, gender, age >65, *MGMT* status, tumor volume using the surgeon’s estimate (due to reduced sample size in analyzable core laboratory submission), and adjuvant radiation, chemotherapy, or immunotherapy ( Table 3).

Disease progression was determined by the treating physician via brain imaging and/or clinical status. PFS estimated by Kaplan-Meier method considered patients “at risk” until they had disease progression or until their last follow-up/death date and were otherwise censored from the analysis. Median is the calculated point where the PFS/OS probability drops below 50%.

All reported *P*-values were two-sided, and a *P*-value <0.05 was considered statistically significant. All statistical analyses were performed using SAS Version 9.4.

## Results

### Overall Demographics and Procedure

A total of 29 newly diagnosed and 60 recurrent adult patients diagnosed with *IDH* wild-type glioblastoma met the criteria for inclusion. Demographic details across both patient cohorts are shown in Table 1. The mean age of patients was 63 and 59 years, and the median KPS at baseline was 85 and 90 for the newly diagnosed and recurrent cohorts, respectively. Recurrent patients had received prior LITT (6.7%), resection (88.3%), radiation (86.7%), and chemotherapy (90%). Roughly half (53.8%) of patients with newly diagnosed glioblastoma (nGBM) initiated treatment with both radiation and temozolomide chemotherapy following LITT and 15.4% of patients also received bevacizumab. Treatment regimens used for patients with newly diagnosed and recurrent diseases starting within 12 weeks of the LITT procedure are shown in Supplementary Tables 1 and 2.

**Table 1.** Baseline Characteristics of Glioblastoma *IDH* Wild-Type WHO Grade 4 Cohort

Characteristics and Measures	Newly Diagnosed (N = 29)	Recurrent Disease (N = 60)	All Subjects (N = 89)
Age, mean (SD), years	62.8 (13.8)	59.0 (11.0)	60.2 (12.0)
Female, No. (%)	9 (31.0)	32 (53.3)	41 (46.1)
Race/ethnicity, No. (%)			
White	25 (86.2)	55 (91.7)	80 (89.9)
Black/African American	3 (10.3)	1 (1.7)	4 (4.5)
Asian	1 (3.4)	1 (1.7)	2 (2.2)
Other/unknown	0 (0)	2 (3.3)	2 (2.2)
Baseline KPS, n/N (%)			
>70	15/22 (68.2)	47/57 (82.5)	62/79 (78.5)
<70	7/22 (31.8)	10/57 (17.5)	17/79 (21.5)
Reason for LITT, n/N (%)			
Non-resectable lesion	14/27 (51.9)	25/59 (42.4)	39/86 (45.3)
Minimally invasive procedure preferred	11/27 (40.7)	25/59 (42.4)	36/86 (41.9)
Other	3/27 (11.1)	8/59 (13.6)	11/86 (12.8)
*MGMT* promoter methylation, No. (%)	5 (17.2)	23 (38.3)	28 (31.5)
Prior therapy (not mutually exclusive)			
LITT ablation	–	4 (6.7)	4 (4.5)
Resection	–	53 (88.3)	53 (59.6)
Chemotherapy	–	54 (90.0)	54 (60.7)
Radiation (not mutually exclusive)	–	52 (86.7)	52 (58.4)
SRS	–	8 (13.3)	8 (9.0)
Whole-brain RT	–	7 (11.7)	7 (7.9)
Local	–	38 (63.3)	38 (42.7)
Procedural EOA (surgeon estimate), n/N (%)			
100	2/27 (7)	18/58 (31)	20/85 (24)
91-99	13/27 (48)	32/58 (55)	45/85 (53)
51-90	10/27 (37)	8/58 (14)	18/85 (21)
Deep seated lesion, n/N (%)	20/34 (58.8)	17/64 (26.6)	37/98 (37.8)
Lesion volume <3 cc, n/N (%)	15/31 (48.4)	20/51 (39.2)	35/82 (42.7)
Lesion volume >3 cc, n/N (%)	16/31 (51.6)	31/1 (60.8)	47/82 (57.3)

Abbreviations: EOA, extent of ablation; *IDH*, isocitrate dehydrogenase; KPS, Karnofsky Performance Scale; LITT, laser interstitial thermal therapy; RT, radiotherapy; SRS, stereotactic radiosurgery.

The primary reason for LITT utilization, as identified by the site, was that the lesion was unresectable using traditional surgical approaches in 52% of newly diagnosed and 42% of recurrent patients. Preference for a minimally invasive procedure was selected in 41% and 42% of patients in the newly diagnosed and recurrent cohorts, respectively. Most tumors treated with LITT in the newly diagnosed cohort were deep-seated (59%) compared with 27% in the recurrent group. Lesion volumes, per physician report, were greater than 3 cc in 51.6% of newly diagnosed patients and 60.8% of recurrent disease patients. Thalamic lesions specifically accounted for 17.6% of the newly diagnosed group and 9.4% of the recurrent group.

### Procedural Safety and Hospitalization Data

In patients with recurrent glioblastoma, 42/60 (70%) had intraoperative biopsy, with 26 showing tumor recurrence, 13 showing mixed tumor and radiation necrosis, and 3 showing radiation necrosis with no active tumor. The mean surgical “skin to skin” time was 4.07 hours for newly diagnosed and 3.29 hours for recurrent tumors. The average number of trajectories per patient was 1.3 + 0.5. Total lase time and total energy applied were available on 52/89 patients. The median lase time was 26 minutes 32 seconds and 25 minutes 8 seconds for the newly diagnosed and recurrent cohorts, respectively. The median total energy applied was 22 250 kJ and 20 840 kJ. The median follow-up time post-procedure for all patients was 268 days. Per core laboratory analysis on 49/89 (55%) patients with evaluable data, the median pre-ablation tumor volume was 7.7 cc (IQR 3.2, 20.3) for newly diagnosed and 8.5 cc (IQR 5.0, 14.0) for those in the recurrent group. There were 7 newly diagnosed and 4 recurrent patients who had sub-total ablations (<100% ablation) while 16 and 22 had total or supra-total ablations, respectively (≥100% ablation). Of the 11 sub-total ablations in the combined cohort, 6 were 90% or more ablated. The univariate analysis with median survival time and percentage ablation, stratified by newly diagnosed and recurrent disease, did not show statistical significance (*P*-values: 0.468 and 0.494, respectively).

The median length of hospital stay was 50 hours (IQR 31.2, 84.2), and 80% of patients were discharged to home. ICU stay post-procedure occurred in 50% of cases for the combined groups. A summary of this data is shown in Table 2. Overall, these findings were commensurate with prior studies of LITT in this patient population.^11^

**Table 2.** Procedural Outcomes and Adverse Events

Characteristics and Measures	Newly Diagnosed (N = 29)	Recurrent Disease (N = 60)	All Subjects (N = 89)
Procedure time, mean (SD), hours	4.07 (1.78)	3.29 (1.41)	3.54 (1.57)
Length of hospital stay, median (IQR), hours	84.0 (43.6, 142.2)	32.3 (30.2, 55.3)	50.0 (31.2, 84.2)
Transferred to ICU post-LITT, n/N (%)	18/28 (64.3)	26/60 (43.3)	44/88 (50)
Discharged to home, No. (%)	19 (65.5)	52 (86.7)	71 (79.8)
Adverse events, No. (%)	6 (20.7)	6 (10)	12 (13.5)
Neurological deficit	3 (10.3)	2 (3.3)	5 (5.6)
Temporary deficit	1 (3.4)	1 (1.7)	2 (2.2)
Permanent deficit	2 (6.8)	1 (1.7)	3 (3.4)
Motor	1 (3.4)	0 (0)	1 (1.1)
Speech aphasia	1 (3.4)	1 (1.7)	2 (2.2)
Seizure	0 (0)	1 (1.7)	1 (1.1)
Edema, symptomatic worsening	1 (3.4)	3 (5.0)	4 (4.5)
Hemorrhage, clinically significant	1 (3.4)	0 (0)	1 (1.1)
Deep vein thrombosis	1 (3.4)	0 (0)	1 (1.1)

Abbreviations: ICU, intensive care unit; IQR, interquartile range; LITT, laser interstitial thermal therapy.

AEs were also similar to prior reports.^24^ AEs occurred in 13.5% (12/89) of patients (Table 2). Twelve AEs were adjudicated as procedure-related by the safety committee. No AEs were adjudicated as NeuroBlate System related. The most frequently reported complication was neurological deficit, occurring in 5.6% (3.4% of which were permanent) and edema (4.5%). Mild or moderate permanent aphasia occurred in 2 patients (2.2%). One patient (1.1%) experienced a permanent motor deficit following ablation of a right frontal lesion abutting the motor strip characterized by left hemineglect, left lower facial droop, and decreased spontaneous movement of the left lower extremity. Two temporary deficits (1 visual and 1 motor) resolved within 30 days of the procedure. One deep vein thrombosis (DVT) occurred and 1 hemorrhage also occurred (Table 2). There were no deaths related to the procedure.

### Progression-Free Survival and Overall Survival

**Figure 2.**
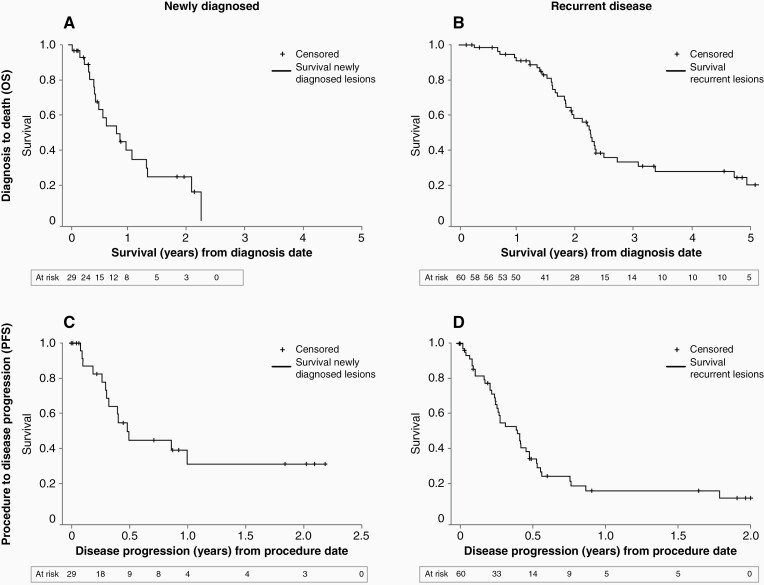
Estimated survival by Kaplan-Meier method—newly diagnosed vs recurrent OS; newly diagnosed vs recurrent PFS. (A) OS from diagnosis to death in the newly diagnosed population; 12-month estimated survival was 40.0%. (B) OS from diagnosis to death in the recurrent disease population; 12-month estimated survival was 91.1%. (C) Time from the LITT procedure to disease progression in the newly diagnosed population; estimated 12-month PFS was 31.5%. (D) Time from the LITT procedure to disease progression in the recurrent population; estimated 12-month PFS was 16.4%. Abbreviations: LITT, laser interstitial thermal therapy; OS, overall survival; PFS, progression-free survival.

Median OS, post-procedure survival, and PFS are shown in Table 4 and displayed in Figure 2. For newly diagnosed patients, an OS of 9.73 months [95% confidence interval (CI): 5.16, 15.91] and a PFS of 5.92 months (95% CI: 3.65, not reached [NR]) were observed. A statistically significant OS advantage was observed in patients with nGBM receiving both radiation and chemotherapy within 12 weeks of LITT (16.14 months, 95% CI: 6.11, NR) vs those who received some of only one treatment modality or no treatment following LITT (5.36 months, 95% CI: 2.14, 7.69). The same advantage was observed in terms of PFS for this same group; 11.93 months (95% CI: 3.65, NR) vs 3.88 months (95% CI: 0.99, 5.92), respectively. Three nGBM patients had a baseline KPS of <60. Two of these 3 patients received no treatment following LITT. Supplementary Table 4 shows survival analysis for nGBM based on KPS, only including those with known baseline KPS >60. In the recurrent group, post-procedure OS was 8.97 months (95% CI: 6.94, 12.36) and PFS was 4.83 months (95% CI: 3.02, 5.82).

**Table 3.** Multivariate Analysis

Variable	Newly Diagnosed		Recurrent Disease		All Subjects	
	Hazard Ratio (95% CI)	*P*-value	Hazard Ratio (95% CI)	*P*-value	Hazard Ratio (95% CI)	*P*-value
Age >65	0.91 (0.2, 4.09) N = 15	.907	0.93 (0.38, 2.31) N = 17	.882	0.93 (0.49, 1.77) N = 32	.828
Female gender	3.38 (0.74, 15.53) N = 9	.117	1.26 (0.57, 2.79) N = 32	.567	1.5 (0.8, 2.79) N = 41	.202
*MGMT*-methylated genetic marker	0.41 (0.02, 6.83) N = 5	.535	0.41 (0.17, 0.95) N = 23	.038	0.43 (0.2, 0.9) N = 28	.025
Tumor volume ≤3 cc	0.88 (0.23, 3.4) N = 12	.851	0.45 (0.18, 1.12) N = 20	.086	0.43 (0.23, 0.81) N = 32	.008
Adjuvant chemotherapy within 12 weeks	0.17 (0.02, 1.31) N = 15	.089	0.39 (0.11, 1.36) N = 42	.139	0.23 (0.1, 0.52) N = 57	<.001
Adjuvant radiation within 12 weeks	0.19 (0.03, 1.43) N = 17	.107	100.34 (6.88, 1462.95) N = 2	.001	0.7 (0.33, 1.48) N = 19	.346
Adjuvant immunotherapy within 12 weeks	– N = 0	–	0.65 (0.19, 2.29) N = 9	.506	0.43 (0.13, 1.43) N = 9	.171

**Table 4.** Median Survival Time

Median Survival (95% CI), Months	Time Months, Average (SD)		
	Diagnosis to Death (OS)	Procedure to Progression (PFS)	Post-Procedure Overall Survival (Procedure to Death)
All subjects (*IDH* wild type)	23.64 (19.17, 27.19)	5.03 (3.42, 5.92)	8.97 (6.94, 11.97)
Recurrent disease	27.19 (23.01, 32.58)	4.83 (3.02, 5.82)	8.97 (6.94, 12.36)
Newly diagnosed	9.73 (5.16, 15.91)	5.92 (3.65, NR^a^)	8.58 (4.50, 14.96)
Chemo + radiation by 12 weeks	16.14 (6.11, NR^a^)* N = 8	11.93 (3.65, NR^a^) N = 7	14.96 (5.88, NR^a^)* N = 8
Chemo/radiation alone, or neither at 12 weeks	5.36 (2.14, 7.69) N = 10	3.88 (0.99, 5.92) N = 7	5.36 (1.58, 7.07) N = 10

Abbreviations: *IDH*, isocitrate dehydrogenase; OS, overall survival; PFS, progression-free survival.

^a^Due to small sample size, the upper bound confidence interval was not reached (NR).

For newly diagnosed glioma: there was a significant difference (*P < .001) from diagnosis to death and procedure to death in tumors treated with vs without combination therapy.

### Functional Assessment

Sixty of 89 patients had baseline KPS recorded. Post-LITT, 7/16 (43.8%) newly diagnosed and 22/44 (50%) recurrent patients had stable or improved KPS over baseline at 1-month follow/up; however, these changes were not significant (*P*-value 0.2502) (Supplementary Table 3). The median KPS at baseline for both groups combined was 90 (IQR 80.0, 90.0) and was 80 (IQR 70.0, 90.0) at both the 1 and 3-month post-procedure timepoints.

## Discussion

*IDH* wild-type glioblastomas are highly infiltrative, surgically incurable tumors. However, maximal safe cytoreduction, through LITT^25,26^ or traditional resection, remains an important mainstay of treatment for these tumors. Treatment options for newly diagnosed and recurrent glioblastoma remain limited, especially for patients with deep-seated and minimally resectable/unresectable tumors. In nGBM, retrospective studies have clearly established that traditional surgical resection of greater than 85% of tumor volume is associated with improved survival.^27,28^ A similar extent of resection relationship has not been established in patients with recurrent glioblastoma although an estimated 25%-30% of patients undergo re-resection at recurrence. In patients not amenable to open craniotomy or for those with unresectable tumors, LITT has become a sought-after treatment option due to the ability to ablate tumor, frequently in deep locations, with a short hospital stay and recovery time, and minimal morbidity.

To provide a comprehensive outcomes analysis of newly diagnosed and recurrent glioblastoma patients undergoing LITT, we carefully identified patients with known *IDH* wild-type status and included patients without molecular *IDH* status only if they were older than 55 years at the time of tumor diagnosis given the low frequency of *IDH* mutation in older patients.^21^ This inclusion of patients with known *IDH* status, or those with the highest probability of having *IDH* wild-type glioblastoma, decreased the overall sample size but heightened our confidence that patients with the same molecularly defined tumor were included. Of note, only those patients with prior diagnoses of histologically confirmed glioblastoma were reclassified using age-related *IDH* status prediction.

LITT was not a replacement for radiation and chemotherapy. As expected, newly diagnosed patients that did not receive post-LITT radiation and chemotherapy had significantly reduced OS vs those who did. Those who received any standard of care radiotherapy with temozolomide (concurrent and/or adjuvant) following LITT had an improved median OS of 16.14 months and a median PFS of 11.93 months (Table 4), both comparable to OS and PFS metrics of patients treated on contemporary clinical trials involving conventional surgical resection (10-21 months, 6-8 months, respectively).^29,30^ Typical OS for glioblastoma patients receiving biopsy only followed by standard of care is 9 months.^7^ It should be noted that these survival comparisons are from literature published prior to the recent 2021 WHO classification guidelines, which makes the comparative benefit derived from this pure *IDH* wild-type cohort even more notable. Thus, in nGBM, LITT followed by standard of care chemoradiotherapy offers an efficacious alternative to traditional surgery for patients who are not candidates for conventional surgical resection.

In recurrent glioblastoma, there is no consensus for the role of re-resection or optimal treatment beyond the need for better therapies.^31^ Many patients with recurrent glioblastoma do not undergo re-resection but instead receive optimal medical therapy with a median post-progression survival of 7-10 months.^32,33^ For those who do undergo re-resection, median post-procedure survival ranges from 5 to 13 months.^33,34^ Of course, patients eligible for re-resection may have a survival advantage simply by being able to tolerate surgery, whether due to age, KPS, or tumor size and location. For the recurrent group, the LAANTERN study does not provide details on the timing of LITT in relationship to the number of prior recurrences and therapies. However, since clinical trials are a first priority consideration for all patients as per NCCN guidelines,^35^ we suspect these data reflect LITT being utilized later in the treatment of individual patients, possibly at the time of secondary or tertiary progression, or beyond. Despite this, the post-procedure OS (8.97 months) for the recurrent group was similar to post-recurrence survival in other studies,^35,36^ including those specifically treating at first progression only.^37,38^ When patients are not eligible for trials due to the number of prior recurrences, lack of trial options, poor KPS, or other eligibility criteria, LITT remains an important treatment option, particularly when LITT can be followed with chemotherapy as also shown in prior reports.^17^

Reasons for LITT selection have expanded beyond simply having an inaccessible lesion. There was a near-equal division in the data for LITT selection between non-resectable lesions and a preference for a minimally invasive procedure. The preference for a minimally invasive option can emanate from various patient needs or concerns and could lead to inclusion of a larger number of patients with worse prognosis or biopsy-only patients.^39^ Thus, patient selection bias may have played a role in the outcomes of patients treated on this study. It would be expected that the majority of patients with the best prognosis would undergo traditional surgical resection per NCCN guidelines. However, patients treated with LITT followed by standard of care therapy in this study fared well based on contemporary studies as comparators.^35^

Patients with imaging changes concerning for tumor progression often undergo biopsy which offers the opportunity to utilize LITT to ablate abnormal tumor tissue and/or treatment-related necrosis. Radiation necrosis occurs in 2.5%-5% of glioma patients following radiation^40^ and can be symptomatic, sometimes necessitating interventions like bevacizumab, which can be associated with high rates of complications.^41,42^ In our study, 22% of recurrent patients (13/60) had a mixture of tumor and necrosis, as often observed in recurrent glioblastoma^43,44^ whereas 5% (3/60) of recurrent patients had pure treatment effect. Although biopsies are subject to sampling error, LITT is effective for the treatment of tumor as well as radionecrosis^45,46^ and should therefore be considered as a cytoreductive treatment modality in the context of recurrent GBM.

LITT has been shown to be safe in a large number of studies,^11,12,14,17,47,48^ which we confirm here. Most AEs were temporary, related to an increase in procedure-related cerebral edema, and resolved within 30 days. Importantly, KPS was maintained in the majority of patients after a small decrease post-procedure.

There are several limitations of this study, including the variability in standard of care practices that an observational registry allows. Registries collect large sample sizes with real-world data across institutions, helping to improve generalizability compared to single-center studies. However, the lack of a defined treatment plan can result in variability in therapy among sites and physicians. Additional data variables would be helpful to understand some of the treatment decisions made in this analysis. For example, granularity regarding the reason why some patients did not receive additional therapy following LITT and understanding at which recurrence patients received LITT would help provide further context in interpreting survival data outcomes. For multivariate analysis, a tumor volume of less than or greater than 3 cc was selected as the cutoff based on the range in physician-reported tumor sizes that happened to be enrolled. Core laboratory EOA data analysis was able to be performed on 49/89 patients who had complete pre and post-procedure imaging submitted. Of the 49 patients, only 11 (7 new and 4 recurrent) were found to have had a sub-total ablation and of these, 6 patients had a 90% or greater ablation (near-total ablation). This lack of sub-totally ablated patients likely contributed to the finding of no statistical significance in the univariate analysis looking at EOA and survival. Post-procedure imaging was collected at any time point from immediately postoperative through 3 days postoperative, creating variation in the amount of post-LITT inflammation and thus variation in EOA estimation. Future volumetric and controlled imaging analyses will be beneficial.

Molecular marker collection was not consistent across all institutions, possibly skewing our understanding of what percentage of patients carried *MGMT* promoter methylation status, which is prognostically significant and a valuable part of survival analysis. Additionally, selection bias concerns surrounding lesion size, functional status, and age are common in prospective neuro-oncology studies, as well as this study, and may limit some broader interpretation of the results. Efforts were made to mitigate these biases in analysis.

Although this study was prospective in its data collection, randomized studies are the primary way to provide level-1 evidence. However, there exist many challenges to designing such studies with an FDA-cleared surgical intervention that is available on the commercial market. In addition to the competing decision by physicians to offer LITT outside of established trials, there are challenges that influence patient and provider interest in enrolling patients into a clinical trial. Given these challenges, an alternative approach is to develop a synthetic control consisting of a contemporary, patient-level data matched control group with patients from the same institutions where these registry data were obtained.

## Conclusion

This prospective multicenter registry study allowed for the analysis of a large, molecularly defined, cohort of patients that has undergone laser ablation. LITT offers an effective cytoreductive approach for patients with newly diagnosed and recurrent *IDH* wild-type glioblastoma. Importantly, its use in newly diagnosed patients who are followed by post-LITT chemoradiotherapy produces a median OS similar to that of patients treated with conventional surgical resection, thus making LITT a viable alternative in patients with inoperable tumors or those not amenable to resection.

## Supplementary Material

vdac040_suppl_Supplementary_Tables

## Notes

**Authorship statement**. Manuscript writing: J.D.G. Manuscript publication planning: J.D.G., A.H.K., S.P., G.R., V.C., and E.C.L. Manuscript review and editing and contribution to the LAANTERN prospective registry database: all authors.
