# Supplementary material for: Efficacy of laser interstitial thermal therapy (LITT) for newly diagnosed and recurrent IDH wild-type glioblastoma
Source: Neurooncol Adv. 2022 Apr 6;4(1):vdac040. doi: 10.1093/noajnl/vdac040 (PMC9122789; doi:10.1093/noajnl/vdac040)
Supplement: vdac040_suppl_Supplementary_Tables [file vdac040_suppl_supplementary_tables.docx]

# Supplementary Figures:

**Supplementary Table 1: Post LITT Medical Management occurring within 12 weeks post LITT Procedure Date**

| **Medical Therapy,** No. (%) | **Newly Diagnosed**  **N=26** | **Recurrent Disease**  **N=51** | **All subjects**  **N=77** |
| --- | --- | --- | --- |
| Chemotherapy (not mutually exclusive) | 15 (57.7) | 44 (86.3) | 59 (76.6) |
| Temozolomide | 15 (57.7) | 11 (21.6) | 26 (33.8) |
| Bevacizumab | 5 (19.2) | 24 (47.1) | 29 (37.7) |
| Lomustine | 2 (7.7) | 11 (21.6) | 13 (16.9) |
| Etoposide | 0 | 1 (2.0) | 1 (1.3) |
| Carboplatin | 0 | 7 (13.7) | 7 (9.1) |
| Other | 1 (3.8) | 17 (33.3) | 18 (23.4) |
| Radiation | 18 (69.2) | 3 (5.9) | 21 (27.3) |
| SRS | 1 (3.8) | 0 | 1 (1.3) |
| Whole Brain RT | 0 | 1 (2.0) | 1 (1.3) |
| Local Radiation | 17 (65.4) | 2 (3.9) | 19 (24.7) |
| Immunotherapy | 2 (7.7) | 9 (17.6) | 11 (14.3) |
| Ipilimumab | 0 | 0 | 0 |
| Nivolumab | 0 | 2 (3.9) | 2 (2.6) |
| Pembrolizumab | 1 (3.8) | 7 (13.7) | 8 (10.4) |
| Atezolizumab | 0 | 0 | 0 |
| Avelumab | 0 | 0 | 0 |
| Steroids | 14 (53.8) | 13 (25.5) | 27 (35.1) |
| Dexamethasone | 14 (53.8) | 11 (21.6) | 25 (32.5) |
| Prednisone | 0 | 2 (3.9) | 2 (2.6) |

Subjects who did not complete at least a 1 month post LITT follow-up visit were excluded

**Supplementary Table 2: Chemotherapy and Radiation Combinations within 12 weeks Post LITT for Newly Diagnosed**

| **Therapy Combination,** No. (%) | **Newly Diagnosed**  **N=26** |
| --- | --- |
| Chemotherapy AND Radiation | 14 (53.8) |
| Chemotherapy |  |
| Temozolomide | 14 (53.8) |
| Bevacizumab | 4 (15.4) |
| Lomustine | 2 (7.7) |
| Radiation |  |
| SRS | 1 (3.8) |
| Whole Brain RT | 0 |
| Local Radiation | 13 (50) |
| Chemotherapy ONLY | 1 (3.8) |
| Temozolomide | 1 (3.8) |
| Bevacizumab | 1 (3.8) |
| Lomustine | 0 |
| Radiation ONLY | 4 (15.4) |
| SRS | 0 |
| Whole Brain RT | 0 |
| Local Radiation | 4 (15.4) |
| No chemotherapy or radiation | 7 (26.9) |

Subjects who did not complete at least a 1 month post LITT follow-up visit were excluded

**Supplementary Table 3: KPS Changes Over Time**

| **Measure** | **Newly Diagnosed** | **Recurrent Disease** | **P-value** | **All subjects** |
| --- | --- | --- | --- | --- |
| **Baseline Score**  (Mean±SD, Median) | 80.5 ± 14.3  85.0 | 84.7 ± 10.5 90.0 |  | 83.5 ± 11.8 90.0 |
| **1 Month** |  |  |  |  |
| Total Score  (Mean±SD, Median) | 72.0 ± 17.0  75.0 | 78.3 ± 14.3 80.0 | - | 76.4 ± 15.4 80.0 |
| No Change | 25.0% (4/16) | 43.2% (19/44) | 0.2502 | 38.3% (23/60) |
| Improvement | 18.8% (3/16) | 6.8% (3/44) |  | 10.0% (6/60) |
| Worsening | 56.3% (9/16) | 50.0% (22/44) |  | 51.7% (31/60) |
| **3 Months** |  |  |  |  |
| Total Score  (Mean±SD, Median) | 74.7 ± 15.0 70.0 | 77.4 ± 14.1 80.0 |  | 76.5 ± 14.3 80.0 |
| No Change | 35.7% (5/14) | 36.1% (13/36) | 0.2819 | 36.0% (18/50) |
| Improvement | 28.6% (4/14) | 11.1% (4/36) |  | 16.0% (8/50) |
| Worsening | 35.7% (5/14) | 52.8% (19/36) |  | 48.0% (24/50) |
| **6 Months** |  |  |  |  |
| Total Score  (Mean±SD, Median) | 66.7 ± 17.8 70.0 | 75.7 ± 15.0 75.0 |  | 73.0 ± 16.2 70.0 |
| No Change | 27.3% (3/11) | 38.5% (10/26) | 0.6128 | 35.1% (13/37) |
| Improvement | 0 | 3.8% (1/26) |  | 2.7% (1/37) |
| Worsening | 72.7% (8/11) | 57.7% (15/26) |  | 62.2% (23/37) |
| **1 Year** |  |  |  |  |
| Total Score  (Mean±SD, Median) | 72.9 ± 21.4 70.0 | 80.0 ± 10.8 80.0 |  | 77.5 ± 15.2 80.0 |
| No Change | 28.6% (2/7) | 25.0% (3/12) | 0.8897 | 26.3% (5/19) |
| Improvement | 14.3% (1/7) | 8.3% (1/12) |  | 10.5% (2/19) |
| Worsening | 57.1% (4/7) | 66.7% (8/12) |  | 63.2% (12/19) |
| **2 Years** |  |  |  |  |
| Total Score  (Mean±SD, Median) | 93.3 ± 5.8 90.0 | 80.0 ± 10.0 80.0 |  | 85.0 ± 10.7 90.0 |
| No Change | 33.3% (1/3) | 50.0% (2/4) | 0.4594 | 42.9% (3/7) |
| Improvement | 33.3% (1/3) | 0 |  | 14.3% (1/7) |
| Worsening | 33.3% (1/3) | 50.0% (2/4) |  | 42.9% (3/7) |

**Supplementary Table 4: Newly Diagnosed Median Survival, KPS>60**

|  | **Post LITT Medical Management (within 12 weeks of LITT procedure date)** | | |
| --- | --- | --- | --- |
| **Time** | **Chemotherapy and Radiation Median Survival (95% CI)** | **Chemotherapy Alone, Radiation Alone, or Neither Median Survival (95% CI)** | **Log Rank P value** |
| Time from Diagnosis to Death OS (months) | 25.18 (5.16, NR*)  N = 5 | 7.91 (4.17, NR*)  N = 4 | 0.020 |
| Time from Procedure to Death (months) | 25.18 (3.65, NR*)  N = 5 | 7.91 (3.48, NR*)  N = 4 | 0.053 |
| Time from Procedure to Progression (months) | 10.29 (2.27, NR*)  N = 6 | 5.92 (1.18, NR*)  N = 3 | 0.461 |

*Due to small sample size the upper bound confidence interval was not reached (NR)
